# Obesity and Childhood Leukemia: Biological, Regional, and Environmental Insights

**DOI:** 10.2174/0118715303359757250213064623

**Published:** 2025-03-10

**Authors:** Marina Costa Fonseca, Vinícius dos S. Carvalho, Giovanna R. Degasperi

**Affiliations:** 1 School of Medicine, Pontifical Catholic University of Campinas, SP, Brazil;; 2 Postgraduate Program in Health Sciences, Pontifical Catholic University of Campinas, SP, Brazil

**Keywords:** Obesity, adipose tissue, childhood cancer, leukemia, environmental factors, metabolic condition

## Abstract

This mini-review aimed to present an outlook on childhood obesity and leukemia in different regions of Brazil and explore a possible association between biological aspects related to adipose tissue and childhood cancer. We discussed environmental factors that may influence cancer development and obesity and present studies showing different prevalences of these diseases in Brazilian regions. Furthermore, we also discussed possible associations of obesity and leukemia considering the biological aspects of adipose tissue associated with disease development. However, future studies are required to determine how environmental factors and obesity contribute to disease development in childhood and adolescence.

## 1 INTRODUCTION

Obesity is a complex metabolic condition currently considered an epidemic in several countries, including Brazil [1, 2]. In addition, it represents a significant public health concern that can affect any age group and gender. According to the World Obesity Atlas, 2022, obesity will affect over 100 million children aged 5-9 and over 150 million adolescents aged 10-19 worldwide by 2030. Obesity has a heterogeneous etiology involving biological, environmental, social, cultural, behavioral, genetic, and developmental factors [2, 3]. Factors, such as access to healthy foods and opportunities for physical activity, are influenced by these conditions [4]. In particular, the change from healthy to ready-to-eat food containing fat and sugar has been observed in the population of Brazil, impacting children’s health [2]. Economic growth and rapid urbanization may be associated with the changes in diet consumption and the lifestyle of many children and adolescents [2, 3]. The availability of healthy foods is often limited in low-income areas, leading to higher consumption of processed and calorie-dense foods [5]. Unequal access to healthy foods and lack of physical activity practices are also significant determinants of obesity [6]. In urbanized areas, the expansion of the use of technologies may contribute to a reduction in physical activities that, when associated with the increase in the consumption of fats and sugars, contributes to overweight and obesity [1], especially when an imbalance in caloric intake and expenditure is evident [2, 7]. It is known that this adverse metabolic state has short and long-term consequences, such as cardiovascular disease, hypertension, dyslipidemia, type 2 diabetes, and cancer [3, 8]. Although most tumors associated with obesity occur in adulthood, the role of obesity in the context of the development and progression of childhood cancers, such as leukemia, has recently been investigated [9, 10]. An association was observed between increased body mass index (BMI) and disease prognosis. Results indicate that increased BMI may be correlated with worse event-free survival (EFS) and relapse in leukemic children [11]. Some hypotheses related to this correlation involve hormones from adipose tissue or the immune response against the tumor, which in the context of obesity appears to be impaired.

Considering that we are experiencing a rise in obesity in children and adolescents and also that obese children diagnosed with leukemia may present worse clinical outcomes, we aim to discuss biological aspects of childhood obesity, which could be associated with leukemia, in addition to presenting some studies showing the prevalence of obesity and leukemia in different Brazilian regions and exploring environmental characteristics in these regions that could contribute to triggering obesity and leukemia.

## 2 METHODS

We briefly summarized the information from articles published in databases, such as Web of Science, Scopus, and PubMed, including original studies and systematic reviews. The inclusion criteria involved all peer-reviewed articles published in English or Portuguese and were limited to obesity and leukemia studies in children and adolescents. Two reviewers (V dos SC and MCF) separately evaluated the studies for information extraction. A third reviewer (GRD) participated in the inclusion or exclusion of articles when necessary. Titles and abstracts were reviewed and publications were selected for reading the summary first. A combination of the following words was used in the search: childhood obesity, childhood leukemia, Brazil, adipose tissue, immune response, adipose tissue hormones, states of Brazil, and environmental factors.

### 2.1 A Brief Overview of Childhood Obesity and Leukemia in Different States of Brazil

Brazil is marked by a sizable territorial extension comprising regions with different socioeconomic and cultural realities that may impact the development of obesity in children and adolescents [12]. The Brazilian Institute of Geography and Statistics (IBGE) published a study revealing that 15% of Brazilian children and adolescents are classified as obese. This data agrees with a recent systematic review pointing out a childhood obesity rate of around 12% in Brazil [13]. Furthermore, the prevalence of the disease increased between 2001-2007 and 2008-2014 [14], and an increase was reported in obesity when we compared low-income with high-income populations in Brazil [15]. Regional variations in the prevalence of childhood obesity were also observed in northern Brazil: 2.6% in Para and 15.8% in Rondonia state [16]. This outlook may reflect socioeconomic and regional differences that affect eating and physical activity habits.

Different studies have been widely conducted on children at schools to understand obesity rates in a specific state of Brazil. We aimed to present the findings of some of them to represent a scenario of childhood obesity. A study carried out in the state of São Paulo on around 3,000 children/adolescents aged 7-18 years, selected from public and private schools, reported that 8.9% of boys and 4.3% of girls were obese. Interestingly, it was observed that the higher prevalence of obesity was associated with travel to school, computer usage, and breakfast consumption [17]. Although including a smaller number of children/adolescents from public and private schools in the municipality of Uberaba, a medium-sized city in the state of Minas Gerais, the prevalence of obesity was 12.5% and 18.0%, respectively, in girls and boys aged 5.6 - 18 years [18]. In a systematic review and meta-analysis, Sbaraini *et al*. [19] reported that the southern regions had the highest prevalence of overweight and obesity among adolescents without differences in prevalence by sex. Furthermore, a lower prevalence was observed in obesity among children in the Northeast compared to children in the south [19]. Metabolic profile was also characterized in some studies [20, 21]. A systematic review and meta-analysis, which included more than 40,000 adolescents enrolled in public and private schools in all Brazilian regions, showed a high prevalence of metabolic syndrome in adolescents [22]. Metabolic syndrome is a component associated with obesity [23]. A cross-sectional analysis of children aged 5 - 12 years from public schools in the semi-arid region and also in the state of Bahia identified metabolic factors, such as triglycerides, reactive C protein, and HDL levels associated with obesity [24]. Parental obesity may prone obese children to exacerbated metabolic disturbances, some of them, related to metabolic syndrome such as insulin homeostasis, dyslipidemia, and inflammation [25].

 In Alagoas (Northeast Brazil), an increased prevalence of overweight was associated with a reduction in the prevalence of stunting [26]. Moreira *et al*. [27], in a study carried out in the semiarid region [state of Alagoas], concluded a high prevalence of overweight in children under five years. It was suggested that being overweight was associated with a short period of breastfeeding and maternal central obesity. In a sample comprising 699 children ranging from 5 to 9 years of age from Feira de Santana (state of Bahia), it was observed that overweight and obese children predominated in middle and high social and economic status groups and that this metabolic condition was associated with parent’s obesity [28].

Childhood leukemia in Brazil is comparable to that observed in developed countries [29]. However, significant regional variations reflect socioeconomic differences and access to health care. The south and Southeast regions have slightly higher incidence rates than the north and Northeast regions. Data extracted from the system of the Federal Government of Brazil, DATASUS, from 2019 to 2022, considering individuals under 15 years of age, showed a higher prevalence of childhood leukemia in the Southwestern region of Brazil, followed by the Northeast [30]. Representing the incidence rate of childhood leukemia in Recife, located in the Northeast region, it was found that from 1998 to 2007, the incidence rate of the disease was stable [31].

Considering these articles, it was found that obesity and leukemia have more prevalence in Southeast Brazil. These differences may reflect characteristics of the Southeast, such as economic aspects, lifestyle, eating behavior, factors related to leukemia, better access to healthcare, and modern diagnosis procedures. Limited access to healthcare and a lower socioeconomic position may make it difficult to establish a leukemia diagnosis. However, to better understand these data, large-scale and ecological studies are important to measure the number of obese children in each region and obese children diagnosed with leukemia. It will contribute to the design of new approaches and treatments to achieve better outcomes in obese children with leukemia.

### 2.2 Childhood Obesity, Leukemia, and Environmental Factors

Obesity has been implicated in the development of different types of cancers, and biological evidence indicates that obesity may also be associated with childhood leukemia, as discussed in this study.

However, studies have reported that environmental factors may be associated with obesity and leukemia. Exposure to fine particulate matter (PM2.5) in the ambient air was found to be significantly associated with childhood obesity [32]. Air pollution exposure during the prenatal period is also associated with childhood obesity, especially in females, which could be explained by higher cord blood levels of leptin and high molecular weight adiponectin - hormones from adipose tissue associated with obesity [33]. Furthermore, air pollution (PM2.5 and NO2) exposure may contribute to the development of type 2 diabetes by altering insulin sensitivity and β-cell function [34]. Interestingly, in a study exploring the association between different environmental exposures and obesity in Chinese youth, it was suggested that air pollution, road environment, and built environment were positively correlated with higher BMI and obesity risk. However, meteorological factors reduced it. One explanation for the association between exposure to these pollutants and childhood obesity may be associated with the fact that exposure alters caloric intake and dietary behavior, favoring fat consumption [35]. Experimental evidence indicated that some environmental chemicals, such as bisphenols or agrochemicals, are associated with obesity [36]. Interestingly, it was observed that human mesenchymal stem cell exposure to 2,4-DTBP led to increased lipid accumulation and expression of adipogenic marker genes in these cells [37].

It was found that exposure to pollutants or air contaminants, such as benzene and nitrogen dioxide, is associated with childhood leukemia risk [38]. In another study, this association with childhood leukemia was found to be closely related to benzene [39]. Regarding childhood leukemia, epidemiologic studies have demonstrated that pesticides are risk factors for this childhood cancer [40]. A recent systematic review and meta-analysis demonstrated a positive association between domestic pesticide exposure and childhood leukemia [41].

Considering the evidence that childhood obesity and leukemia may be associated with environmental factors, it is important to adopt strategies to prevent children's exposure to these agents. New studies are important to design how these agents or pollutants lead to leukemia and obesity and whether the same mechanisms are responsible for both diseases.

### 2.3 Biological Aspects of Childhood Obesity and Leukemia

Several factors can associate childhood obesity with the development of leukemia at this stage of life [42]. However, studies are still seeking to elucidate the mechanisms that specifically show how fat can contribute to hindering disease development.

Adipose tissue in the metabolic condition of obesity, in addition to other molecules, produces pro-inflammatory cytokines, such as tumor necrosis factor-alpha (TNFα), interleukin-6 (IL-6), and IL-β, thus promoting a state of chronic inflammation [43]. Adipose tissue also produces leptin and adiponectin, hormones that could be associated with childhood cancer progression [44]. Hypoadiponectinemia is a condition that has been observed in the development and relapse of ALL. Adiponectin effects are mediated by AMP-activated protein kinase (AMPK), which suppresses leukemic cell activity by stimulating the tumor suppressors p53 and p21. Low adiponectin levels in obesity may reduce the activity of tumor suppressors, which may promote the development of ALL [45].

Leptin may also have an important role in leukemia. Leptin also functions as a growth factor for some tumor cells [46]. Increased circulating leptin is present in obese individuals, and its increased levels are also associated with the risk of developing and progressing cancer, such as endometrial, breast (post-menopausal), ovarian, and thyroid cancer, among others [47-49]. This risk may be related to the action of leptin stimulating the proliferation of tumor cells or even negatively modulating immune responses against the tumor. The main antitumor response of adaptive immunity is natural killer and CD8^+^T cells that have a cytotoxic function against tumor cells [50]. Both in the metabolic condition of obesity and the tumor microenvironment, CD8+ T cells can acquire an exhaustion profile, which impairs their effector immune responses, proliferation, and, therefore, their performance in immune responses [51, 52]. Regarding NK cells, it has been shown that obese individuals present a low number [53] with lower cytotoxic activity when compared to non-obese [54, 55].

In addition to leptin, other hormones, such as insulin and IGF-1, may exert mitogenic effects [56]. Insulin exerts mitogenic effects through its signaling pathway that is dysregulated in ALL. This signaling includes overexpression of PI3K/Akt and STAT3 in lymphoblasts, which is associated with chemotherapy resistance and poor prognosis [57].

**Fig. (1):**
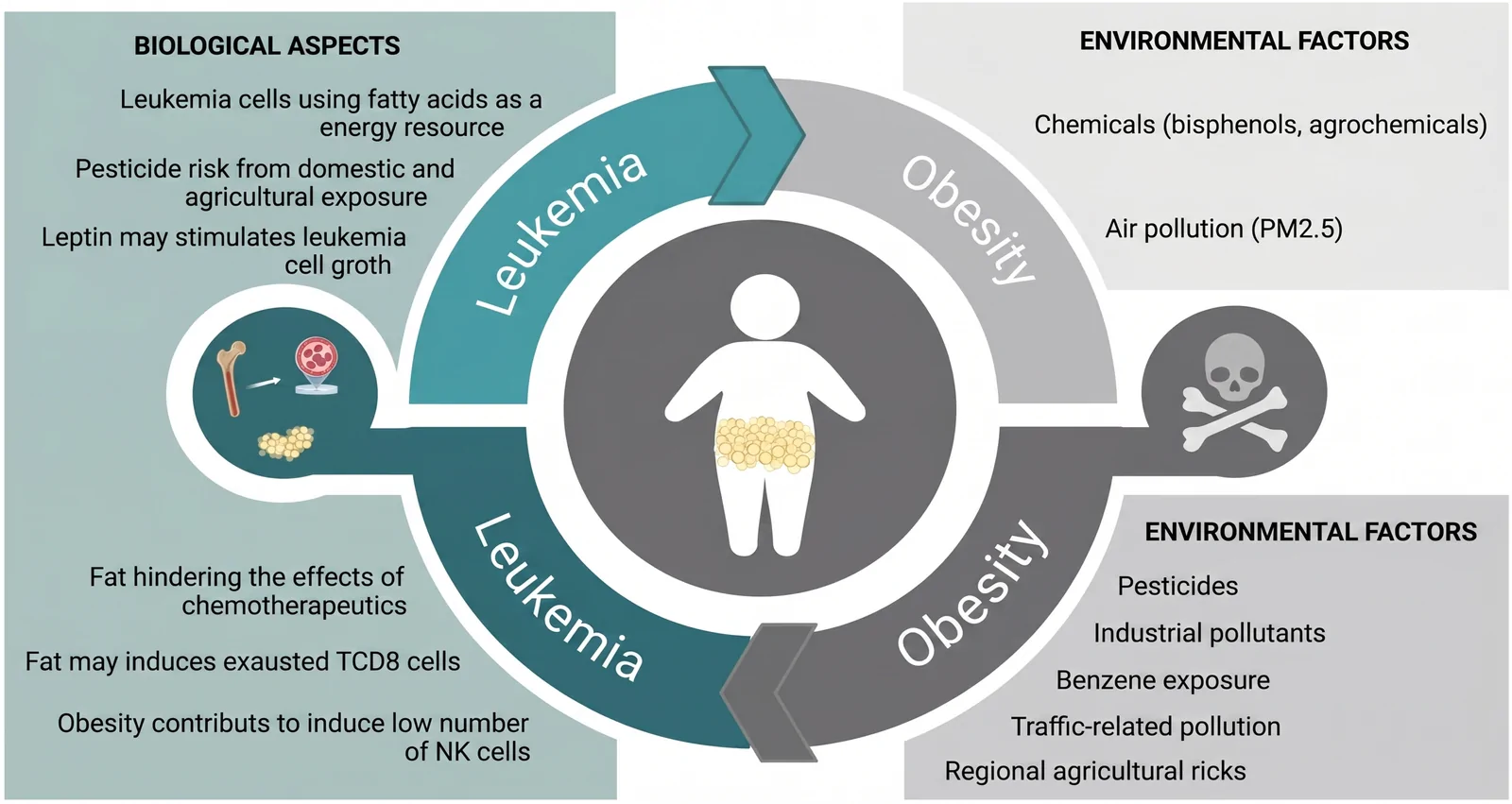
Relationship between leukemia and obesity. The figure illustrates the biological aspects associated with obesity and leukemia as well as the environmental factors that are associated.

Adipose tissue can also function as a reservoir for leukemic cells. Adipocyte stromal cell-derived factor 1-α (SDF-1α), or cysteine-X-cysteine ​​(C-X-C) motif chemokine 12 (CXCL12), has been shown to promote leukemia cell migration into adipose tissue [58] in addition to sequester chemotherapeutic agents, including anthracyclines, daunorubicin, and doxorubicin [59]. Obesity negatively impacts the efficacy of multiple chemotherapeutic agents used in ALL therapy and may also provide energy substrates for leukemic cells. During co-cultures of leukemia cells and bone marrow adipocytes, an overexpression of lipogenic enzymes has been observed in these adipocytes, along with an increase in the expression of fatty acid transporters, such as fatty acid-binding protein-4 [60]. This observation suggests that leukemic cells can use fatty acids as an energy source for proliferation, and adipose tissue appears to be a niche that favors the development and maintenance of the disease [61] (Fig. **1**).

## CONCLUSION

An ecologic investigation should be performed to explore the geographic distribution of childhood obesity and leukemia incidence between Brazilian regions and whether environmental elements specific to different states could be common triggering factors for both diseases. The studies presented in the article have used different age groups and statistical methods. The biological aspects strongly suggest an association between adipose tissue in obesity and leukemia development. However, more experimental and clinical studies are necessary to better understand how adipose tissue contributes to leukemia development in obese compared to lean children.

## AUTHORS’ CONTRIBUTIONS

The authors confirm their contribution to the paper as follows: study conception and design: GD; draft manuscript: MCF and VSC. All authors reviewed the results and approved the final version of the manuscript.

## LIST OF ABBREVIATIONS

AMPK: AMP-Activated Protein Kinase
ALL: Acute Lymphoblastic Leukemia
AML: Acute Myeloid Leukemia
BMI: Body Mass Index
CLL: Chronic Lymphocytic Leukemia
CXCL12: Cysteine-X-Cysteine Motif Chemokine 12
DATASUS: Department of Informatics of the Unified Health System
EFS: Event-Free Survival
HDL: High-Density Lipoprotein
IBGE: Brazilian Institute of Geography and Statistics
IGF-1: Insulin-like Growth Factor 1
IL: Interleukin
NK: Natural Killer (cells)
NO2: Nitrogen Dioxide
PM2.5: Particulate Matter with a diameter of 2.5 micrometers or smaller
SDF-1α: Stromal Cell-Derived Factor 1-Alpha
STAT3: Signal Transducer and Activator of Transcription 3
TNFα: Tumor Necrosis Factor-Alpha
